# Art and Disease

**Published:** 1894-02-24

**Authors:** 


### Art and Disease.

### VI.—HEALING OF THE SICK.

Nothing is too loathsome to be introduced into the scenes from the lives of the saints in order to heighten the effect of their saintliness. Saint Elizabeth has been specially singled out by two celebrated artists as a centrepiece for exceptionally unpleasant realism. In Murillo's great picture at Madrid of this saint she is represented treating with her own hands a child unmistakeably afflicted with ringworm; the irregular bald patches on the head betray the child's malady, and another child violently scratching his head close by is awaiting his turn of treatment. In a picture preserved at Munich of "Saint Elizabeth Feeding the Lepers," by Hans Holbein the Elder, the patients who are grouped round her reveal so realistic a study of this disease as to have attracted the attention of the German physician, Dr. Virchow. He finds in them an exact image of leprosy such as it existed in Germany towards the end of the thirteenth century, and perhaps at Augsburg. As Holbein was born in that place, and remained there during a great part of his life, and as there were three hospices for leprosy in the town, it seems probable that the patients in one of these served as models for the picture.

Even more remarkable from a pathological point of view is the work of Albert Dürer depicting the healing of the lame man at the Temple Gate by Saint John and Saint Peter. Of this work M. Charcot notes: "The diagnostic of the suppliant's malady is remarkably easy. He is simply a leper, attacked by a mixed form of the disease. On the face, and principally on the lips, may be recognised the nodosities of tuberculous leprosy, while the rest of the body bears the marks of atrophic leprosy. . . . The arms are emaciated to the last degree, and the hands are contracted." The left hand assumes a special pose, and both that and the arms reveal the most evident marks of muscular atrophy, first defined and described by Duchenne. The attitude of the hand reveals the unequal invasion of the atrophy, and exactly corresponds to what Duchenne has carefully described as the consequence of the atrophy of the little muscles lodged between the intermetacarpian spaces. "It is most interesting," M. Charcot concludes, "to observe art preceding science, and Albert Dürer, in copying a leper, giving not only an exact image of leprosy, but formulating in an absolutely precise manner, in the year 1513, the morphological characteristics of a muscular alteration which a specialist should only regularly describe three centuries later."

We need not linger over the representations of the Plague. It has given rise to many pictures of amazing dramatic force, but they are hardly such as add to the gaiety of nations, nor, which is more important in this connection, do they exhibit much real acquaintance with the symptoms of the disease. There were obvious reasons against studies from the life in this subject. Studies of boils and blains there are in plenty. Babies clutching the breasts of dead mothers, assistants holding their noses as they approach the sick, doctors dying in the discharge of their duties, dead bodies in abundance.

Saint Roch, who died himself of the plague after tending numerous cases, is usually represented as attacked by some of the symptoms, as a general rule he is pointing to some purely conventional "gavacciolo" on his thigh. The more ambitious plague pictures are (happily) not often more realistic.

Two or three interiors of hospitals have served as subjects for the mediæval Italian artists, and present many points of interest in view of the scanty information obtainable about such institutions at that epoch. A fresco of Taddeo di Bartolo about the end of the fourteenth century represents the hall of an infirmary at the moment when the doctors are going their rounds. Three patients only are visible among a perfect crowd of doctors and attendants. An emaciated figure in the foreground is undergoing the process of a bath, not much to his satisfaction, to judge by the expression of his face. Another patient who has just been brought in on a litter is being raised up to undergo the examination of two doctors, who are discussing his case at a short distance. The third invalid is apparently fast asleep in his bed, with a shelf holding a water bottle and medicines over his head. The scene is rather more animated than would be thought quite seemly in the present day, and the confusion is increased by the pugnacity of the hospital cat, who is violently attacking an intruding cur. A miniature of the fifteenth century shows a room in the Hôtel Dieu, where the patients are represented, two in each bed, neatly tucked up, with an occasional bare arm lying outside. Luca della Robbia, among the works of mercy with which he has ornamented the façade of the hospital at Pistoia, has modelled one from "The Care of the Sick." "The two extremities are occupied by two patients in bed. The one on the left is a fever patient. He is raising himself with difficulty, his head falls forward, his face expresses suffering and exhaustion, his half-open mouth betrays oppression. This physiognomy may be recognised from its being encountered, one might almost say, at every step in our hospitals. Near him the doctor, still wrapped in his cloak, is feeling his pulse in a happily-rendered attitude of calm, dignity, and reserve. He has drawn off his gloves for this purpose, which he holds in his left hand." *

Among minor scenes of healing the operation of bleeding is one of the most popular in art, as it was in all probability by far the most frequently performed in those days. Among the Dutch painters consultations between doctor and patient are a favourite subject. There is much opening for display of character and detail in such pictures, but they are rarely such as one would care to possess. In modern art the bedside study has become entirely banal and sentimental. English artists recoil with wholesome distaste from the repulsive, and the representation of suffering can be justified only by the most scrupulous adherence to truth in the carrying out of some high design.

* "Les Difformes et les Malades dans l'Art." (J. M. Charcot and P. Richey.)

